# Polyallylamine Binds to Aβ Amyloid and Inhibits Antibody Recognition

**DOI:** 10.3390/ijms25063112

**Published:** 2024-03-07

**Authors:** Yusuke Tsuchie, Soichiro Kusuda, Haruka Kawabe, Wakako Mori, Mikael Lindgren, Yutaka Watanabe, Tamotsu Zako

**Affiliations:** 1 Department of Chemistry and Biology, Graduate School of Science and Engineering, Ehime University, 2-5 Bunkyo, Matsuyama 790-8577, Ehime, Japan; 2 Department of Chemistry, Faculty of Science, Ehime University, 2-5 Bunkyo, Matsuyama 790-8577, Ehime, Japan; 3 Department of Physics, Faculty of Natural Sciences, Norwegian University of Science and Technology (NTNU), Gløshaugen, Realfagsbygget, 7490 Trondheim, Norway

**Keywords:** functional amyloids, polymer coating, polyallylamine, thioflavin T assay, detection by antibody

## Abstract

Protein amyloids have attracted attention for their application as functional amyloid materials because of their strong properties, such as high resistance to chemical or biological degradation, despite their medical issues. Amyloids can be used for various applications by modifying the amyloid surface with functional materials, such as proteins and polymers. In this study, we investigated the effect of polyallylamine (PAA), a functional cationic polymer as a candidate for amyloid modification, on the amyloids formed from amyloid β (Aβ) peptide. It was demonstrated for the first time that PAA can bind to Aβ amyloids through fluorescence observations and the quenched emission from the tyrosine at site 10 near the fibrillogenic core. These results suggest that PAA could be used to develop new functional amyloids. However, notably, coating Aβ amyloid with PAA could affect conventional amyloid detection assays such as thioflavin T assay and detection using antibodies. Thus, our results also indicate that consideration would be necessary for the analysis of functional amyloids coated with various polymers.

## 1. Introduction

Several proteins form amyloid fibrils that are associated with neurodegenerative diseases, such as Alzheimer’s and Parkinson’s disease [1]. To date, more than 20 proteins, including amyloid β (Aβ) and α-synuclein, that are involved in amyloid diseases have been identified [2]. These amyloids typically exhibit rigid and unbranched structures with diameters of 10–20 nm and lengths of up to several micrometers. They also commonly show cross-β structure where β-strands are aligned perpendicular to the long axis of amyloids [3]. These amyloids are usually detected by thioflavin T (ThT) fluorescent dye, which is known to bind to accumulated β-sheets [4], or by using antibody staining [5,6,7].

While the medical aspects of protein amyloids have been well studied, increasing attention is on their application as natural and artificial functional amyloid materials by taking advantage of their strong properties, such as high resistance to chemical or biological degradation [8,9,10]. As natural materials, amyloids are utilized in living species for hormone storage [11], nitrogen catabolism [12], and biofilms [13]. These functions could be related to the well-defined cross-β structure of amyloids [8].

Artificial functional amyloids have also been developed for various applications such as scaffolds for cell cultures, organic photovoltaics, drug delivery, and catalysts [8,10]. For example, amyloids formed from the combination of a fragment from the amyloidogenic protein transthyretin and the RGD (Arg-Gly-Asp) sequence enhanced cell growth and differentiation [14]. It was also shown that insulin amyloids could be used for cell adhesion and proliferation [15,16]. Amyloids can be used for optoelectronic applications by modifying the amyloid surface with functional materials, such as proteins and polymers [9]. For example, hybrid nanowires created by coating amyloids made from β-lactoglobulin with titanium dioxide (TiO_2_) and polythiophene could work as photovoltaic devices with excellent power conversion efficiency, making them promising candidates for use in hybrid solar cells [17]. Functionalization of the amyloid surface was achieved using a biotin–streptavidin system; the amyloids were modified with biotin through co-incubation with a protein peptide and biotin, and labeled streptavidin was attached to the amyloids via biotin–streptavidin interactions [18,19]. Streptavidin can be labeled with a variety of substances, such as gold nanoparticles, fluorescent dyes, and enzymes, resulting in amyloids with various activities such as conductivity [20,21], sensing [22], and catalysis [23].

In this study, polyallylamine (PAA), a functional cationic polymer consisting mainly of a single amine, was used to modify amyloids. PAA variants can be used for various purposes such as a gene delivery reagent [24,25] and a reagent to protect insulin from enzymatic degradation [26], suggesting that PAA is a promising functional polymer. Herein, we hypothesized that amyloids with a negative charge would bind to PAA due to the positive charge of PAA, and investigated the effect of PAA on Aβ amyloids, which are negatively charged under neutral conditions.

Herein, we demonstrated for the first time that PAA could bind to Aβ amyloids, suggesting that PAA could be used to develop new functional amyloids. However, notably, coating of Aβ amyloids with PAA could affect conventional amyloid detection assays such as ThT assays and detection using antibodies. Thus, our results also indicate that consideration would be necessary for the analysis of functional amyloids coated with various polymers.

## 2. Results and Discussion

### 2.1. Effect of PAA on the Aβ Amyloid

**Figure 1:**
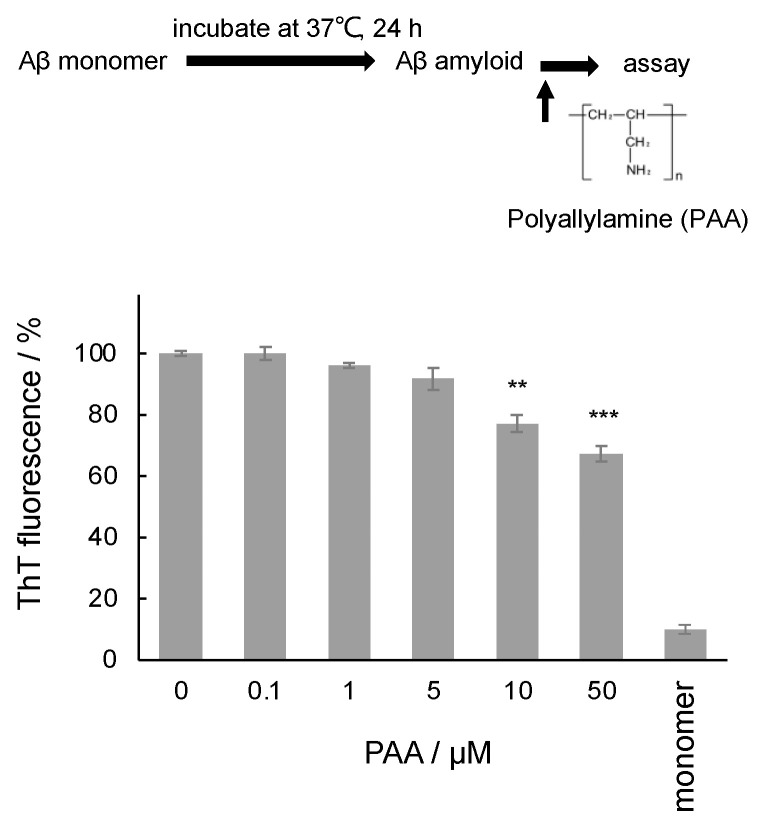
ThT fluorescence assay of Aβ amyloids incubated with 0–50 µM PAA. After Aβ amyloid formation by incubation at 37 °C for 24 h, 0–50 µM PAA was added and incubated at room temperature for 5 min. The ThT fluorescence intensity of the solution including 2.5 µM Aβ42 and 20 µM ThT was measured at 490 nm. The intensities were normalized to the sample without PAA as 100%. The average values of three samples are plotted (**, *p* < 0.01, ***, *p* < 0.005).

First, we investigated the effect of PAA on Aβ amyloids using the ThT fluorescence assay, which can estimate the accumulated β-sheet structure of amyloids by specific binding [4]. PAA was added after the incubation of Aβ42 for the amyloid formation. As shown in Figure 1, ThT fluorescence intensity was similar after the addition of PAA, indicating that PAA had no significant effect on the structure of the amyloids at low concentrations. At higher PAA concentrations, the ThT intensity was slightly decreased: approximately 80% and 70% compared with the intensity without PAA after adding 10 µM and 50 µM PAA, respectively. Since no degradation was observed for the Aβ amyloids incubated with PAA in the Native PAGE analysis (Figure S1a), these results suggest that PAA may bind to Aβ amyloids and weakly inhibit the binding of ThT to the amyloids.

**Figure 2:**
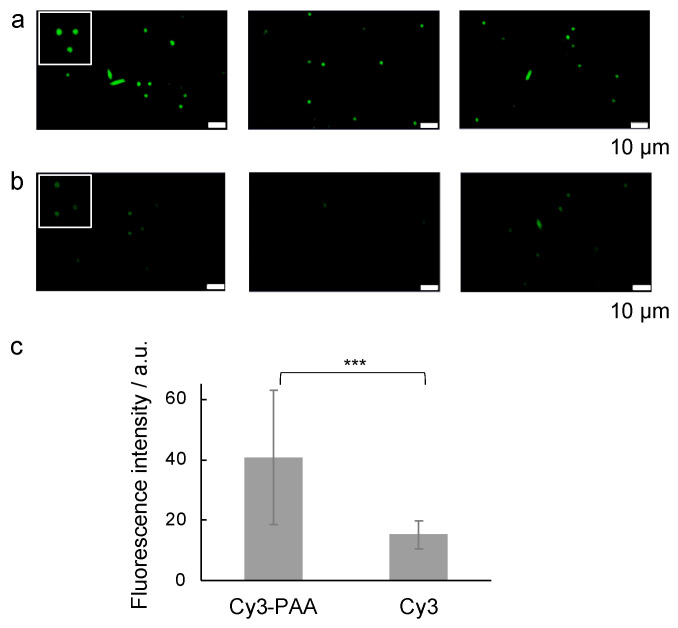
Fluorescence microscopy image of Aβ amyloids incubated with Cy3-PAA. (**a**) Aβ amyloid incubated with Cy3-PAA. Scale bars represent 10 µm. (**b**) Aβ amyloid incubated with Cy3. Scale bars represent 10 µm. The insets are enlarged partial images. (**c**) Fluorescence intensities obtained from (**a**) and (**b**) (***, *p* < 0.005).

To confirm the binding of PAA to Aβ amyloids, Cy3 fluorescent dye-modified PAA (Cy3-PAA) was incubated with Aβ amyloids for fluorescent microscopy observation: the amino group of PAA was modified with Cy3 dye that had N-hydroxy succinimide ester groups. As shown in Figure 2a, fluorescence was observed for Aβ amyloid samples incubated with Cy3-PAA. Importantly, the fluorescent intensities were significantly weaker for Aβ amyloid samples incubated with Cy3 only (Figure 2b,c), supporting that fluorescence was obtained via the binding of PAA to Aβ amyloids and not by non-specific binding of Cy3 dye. These results suggest that PAA can bind to Aβ amyloids.

To further verify PAA’s association with Aβ amyloids, quenching experiments were carried out. Tyrosine absorbs in the 270–280 nm range, typically emits in the range of 300–310 nm, and is commonly used for FRET [27,28] and other quenching experiments in protein chemistry and related fields. For example, tyrosine can intercalate with DNA, resulting in static quenching [29]. The Aβ42 peptide has a single tyrosine residue at position 10. Tyrosine FRET measurements have been used to investigate the role of cholesterol in Aβ peptide interactions with bilayers [30]. Upon adding a suitable acceptor that absorbs in the emission range of the donor, FRET can occur provided the distance is in a suitable range (typically 50–150 Å), and concomitantly, the fluorescence of the donor decreases [27].

**Figure 3:**
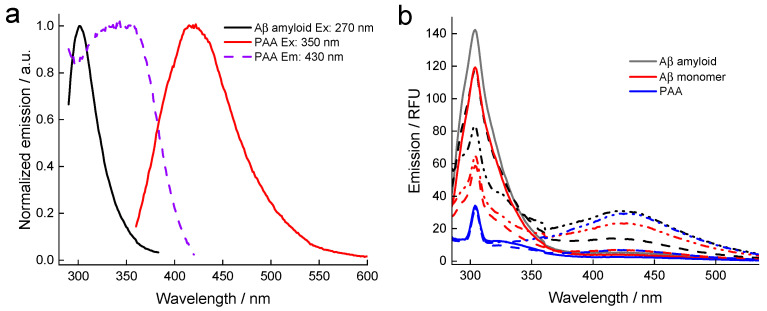

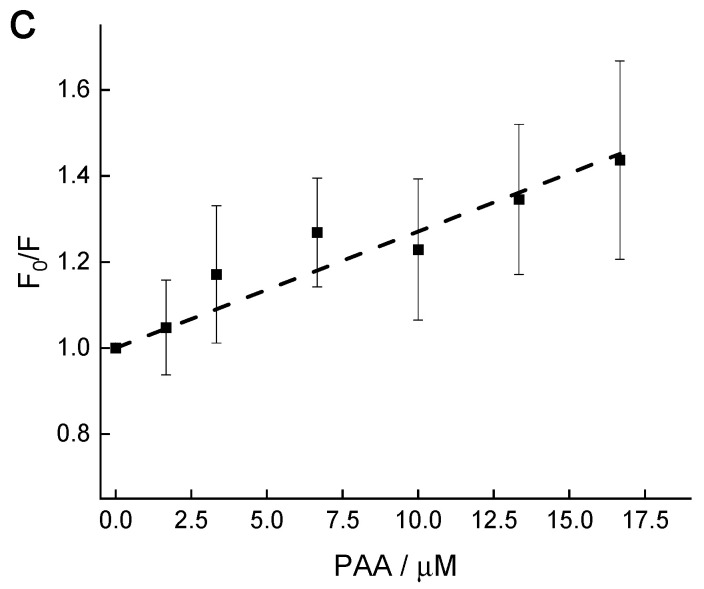
(**a**) Tyrosine emission of the Aβ amyloid (λ_ex_ = 270 nm; solid black) along with the excitation (λ_em_ = 430 nm; dashed blue) and emission of PAA (λ_ex_ = 350 nm; solid red). The spectra have been normalized for easy comparison of peak positions. (**b**) Representative emission spectra at selected concentrations of fluorescence-quenching assays (λ_ex_ = 275 nm) with Aβ amyloid + PAA (black), Aβ monomer + PAA (red) and PPA only (blue) used for baseline correction (solid lines: 0 μM PAA; dashed lines: 3.4 µM PAA; dash-dotted lines: 16.7 μM PAA). (**c**) Stern–Volmer plot obtained as described in the text. The dashed line is a linear fit giving a Stern–Volmer constant K_SV_ = (27 ± 2.1) × 10^3^ mol^−1^ L.

PAA absorbs in the UV range and has a relatively broad emission around 430 nm. The excitation and emission spectra of PAA along with the tyrosine emission of the Aβ amyloid are plotted in Figure 3a, showing the overlap of Aβ emission and PAA absorption, the latter represented by its excitation spectrum. Thus, FRET might be the origin of a quenching process, although a static mechanism cannot be excluded. Quenching assays were prepared by varying the PAA concentration, and representative spectra are shown in Figure 3b for the Aβ amyloid and monomer. As expected, the emission of the tyrosine is strongest for the Aβ amyloid (black lines) as the site in position 10 is near to the fibrillogenic core [31], protected from solvent-induced relaxation. Thus, the emission of the more exposed Aβ monomer (red lines) is substantially weaker (Figure 3b). The emissions of PAA only are also shown and increase substantially for the 17 µM concentration, being similar to the two Aβ cases in amplitude. These also all contain a clear contribution from the solvent Raman scattering peak at 304 nm, and a similar signal is buried under the tyrosine emission of the Aβ cases, as judged from the line shape at low PAA concentrations. Upon increasing the PAA concentration, the emission of the amyloid decreases gradually, owing to quenching, possibly by an FRET mechanism with PAA acting as an acceptor at some fixed distance from the buried tyrosine site. For the flexible monomer, on the other hand, there is also a dramatic quenching for the lowest PAA concentration (3.4 µM) since the less-buried tyrosine of the monomer is more exposed to all of the PAA polymers dispersed in the solvent, a situation very different from the tyrosine fixed in the Aβ fibrillogenic core.

To analyze the quenching of the Aβ amyloid, the PAA-only spectra were first subtracted to remove the background in terms of the disturbing Raman scatter and the PAA emission. The resulting emission spectra were thereafter integrated in the range of 285–360 nm, and a conventional Stern–Volmer plot in terms of F_0_/F was plotted vs. PAA concentration (Figure 3c). The fitted linear relationship is indicative of a quenching process that could possibly be attributed to FRET owing to the spectral relationships between Aβ amyloids and PAA as discussed above, but a static quenching mechanism cannot be ruled out; perhaps both are in operation. We note that the fitted Stern–Volmer constant (Figure 3c), K_SV_ ≅ 27 × 10^3^ mol^−1^ L, is approx. 70% higher than for pristine tyrosine intercalated within DNA following a static quenching process [29]. It was not possible to make a meaningful Stern–Volmer plot based on the Aβ monomer data obtained in the same manner.

**Figure 4:**
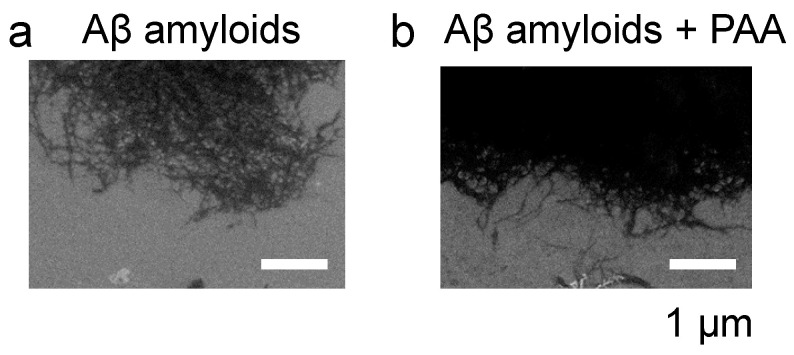
SEM analysis of Aβ amyloids incubated with PAA. (**a**) Aβ amyloids only; (**b**) Aβ amyloids incubated with 50 μM PAA. Scale bars represent 1 µm.

The structures of the amyloids in the absence and presence of PAA were observed using SEM (Figure 4). As shown in the figure, no significant difference was observed between Aβ amyloids alone (Figure 3a) and Aβ amyloids incubated with 50 µM PAA (Figure 4b), suggesting that PAA has no effect on the structure of Aβ amyloids.

**Figure 5:**
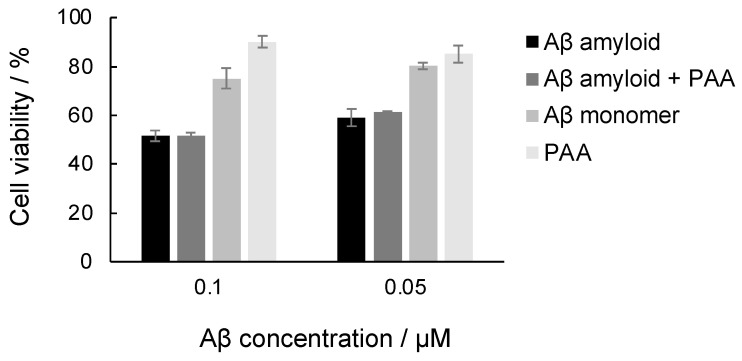
Cytotoxicity of Aβ amyloids incubated with PAA. The Aβ amyloid samples (0.1 µM Aβ with 0.02 µM PAA and 0.05 µM Aβ with 0.01 µM PAA) were added to PC12 cells and incubated at 37 °C for 24 h, and the cell viability was assessed with the MTT assay. The viability for cells exposed to PBS was used as the 100% viability control.

Taken together, the results presented above indicate that PAA spontaneously binds to Aβ amyloids. To further examine the effect of PAA on Aβ amyloids, the cytotoxicity of Aβ amyloids incubated with PAA against PC12 cells using the MTT assay was investigated (Figure 5). As shown in the figure, both Aβ amyloids and Aβ amyloids incubated with PAA showed similar cytotoxicity. In contrast, PAA-only and Aβ monomer samples were less toxic. This result indicates that PAA has no effect on the cytotoxicity of Aβ amyloids.

### 2.2. Effect of PAA on the Binding of Antibody to the Aβ42 Amyloid

**Figure 6:**
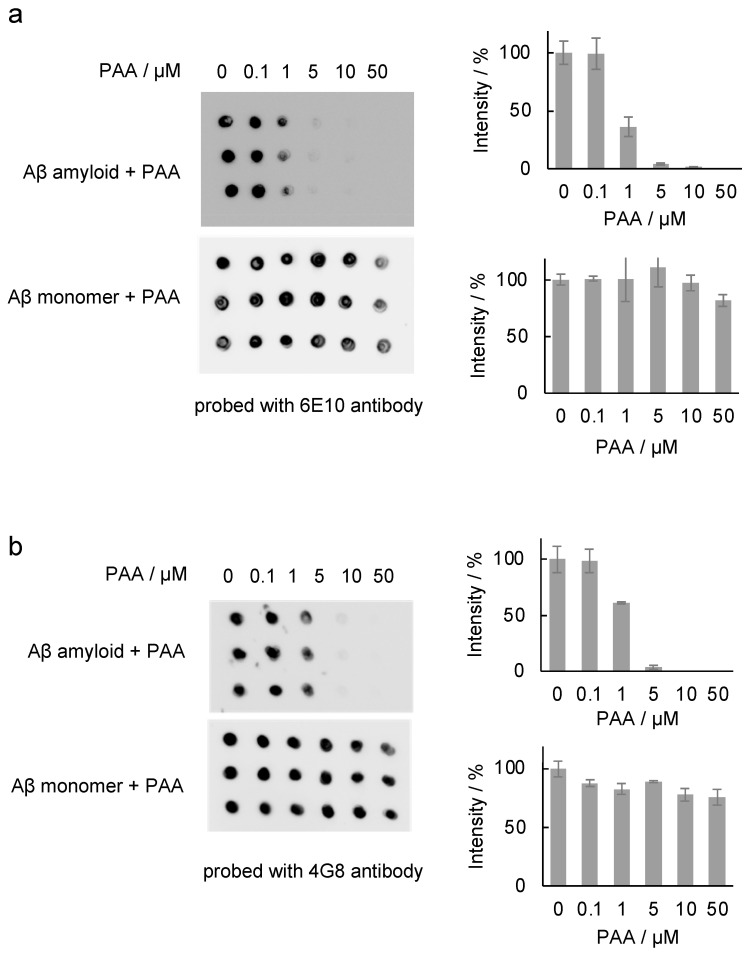
Dot blot assay of Aβ amyloids with or without PAA. Dot blot images of Aβ amyloids incubated with 0–50 µM PAA (upper panels) and Aβ monomers incubated with PAA (lower panels) using antibodies 6E10 (**a**) and 4G8 (**b**) are shown. Graphs show the averaged intensities obtained from the dot blot images.

Antibody analyses are important in amyloid studies [5,6,7]. Thus, the effect of PAA on antibody binding to Aβ amyloids incubated with various amounts of PAA was investigated by the dot blot assay using an Aβ antibody (6E10) that is reactive to amino acid residues 1–16 (Figure 6a). As shown in the figure, the binding of 6E10 to the Aβ monomer was similar even in the presence of PAA, indicating that PAA itself does not interfere with the binding property of 6E10 (Figure 6a, lower). In contrast, the amount of 6E10 bound to the Aβ amyloids decreased in a PAA dose-dependent manner (Figure 6a, upper). This result suggests that PAA may interfere with antibody binding to Aβ amyloids. To confirm this observation, the effect of another Aβ antibody (4G8), which is reactive to amino acid residues 17–24 of Aβ containing the core sequence “KLVFF”, which plays an important role in amyloid formation [32], was investigated (Figure 6b). As shown in the figure, the result was similar to that using the 6E10 antibody: the binding of 4G8 to Aβ amyloids was hindered by PAA, while binding to the Aβ monomer was unchanged in the presence of PAA, supporting our hypothesis that PAA may interfere with the recognition of Aβ amyloids by antibodies.

**Figure 7:**
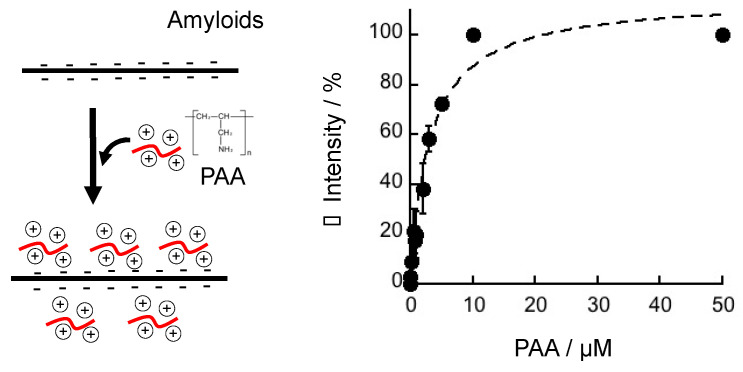
Estimation of affinity between PAA and Aβ amyloids using changes in antibody recognition of Aβ amyloids incubated with PAA. The change in the intensities from the blank sample without PAA in the dot blot images using the 6E10 antibody (Figure S2) was plotted. The average values of three dots are shown. Data were fitted using the equation (△ Intensity = I_∞_ × [PAA]/(K_D_ + [PAA])) and the dissociation constant (K_D_) was calculated to be 3.2 ± 0.55 µM.

The difference in the effect of PAA on the antibody recognition between Aβ amyloids and monomers could be attributed to the difference in surface charge between Aβ amyloids and monomers. The stacked β-sheets have charged sites, whereas the monomers do not have strongly charged regions [33]. Thus, it is plausible that PAA binds to Aβ amyloids via electrostatic interactions. Changes in the antibody recognition were used to estimate the affinity between PAA and Aβ amyloids (Figure 7 and Figure S2). The K_D_ value was estimated to be 3.2 ± 0.55 µM.

### 2.3. Effect of PAA during the Incubation of the Aβ Sample

**Figure 8:**
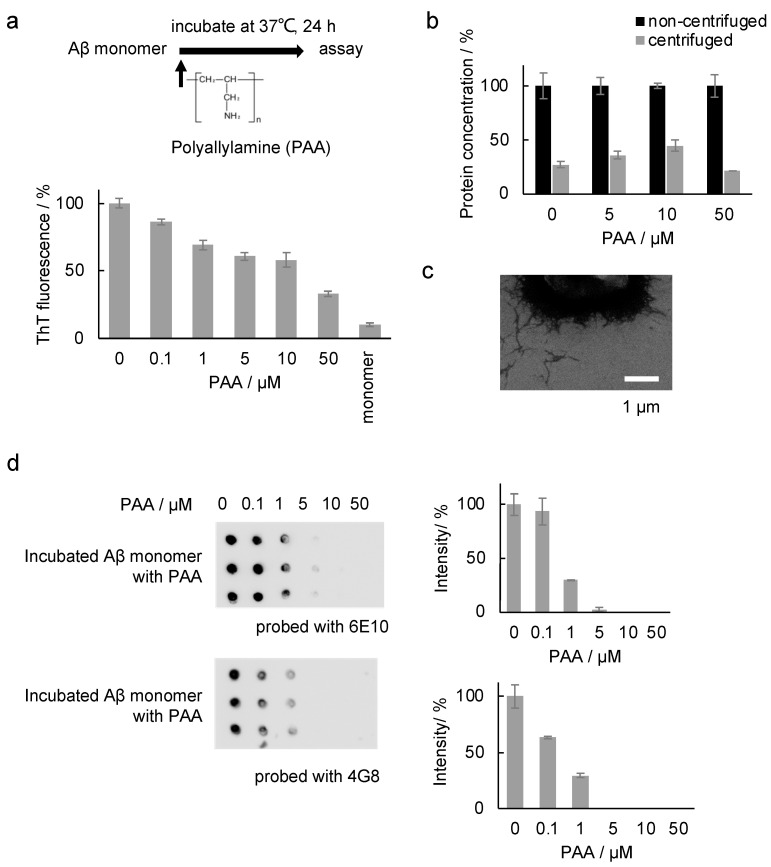
Aβ42 incubated with 0–50 µM PAA. Aβ monomer samples were incubated with or without PAA for further analysis: (**a**) ThT assay; (**b**) protein concentration in the supernatant after centrifugation. The supernatant protein concentration was normalized to the non-centrifuged sample protein concentration as 100%. (**c**) SEM analysis and (**d**) dot blot assay.

Next, to examine the effect of PAA on the amyloid formation of Aβ, PAA was added to the Aβ monomer solution and incubated at 37 °C for 24 h for the following analyses (Figure 8a). Notably, the ThT intensities after incubation decreased in a PAA dose-dependent manner (Figure 8a), suggesting the inhibition of amyloid formation by PAA. To confirm this, the Aβ concentration of the supernatant samples after centrifugation was measured (Figure 8b). The result indicates that the amount of unaggregated Aβ samples in the supernatant was unchanged by the addition of PAA. No increase in the amount of Aβ monomers and soluble species of the Aβ samples incubated with PAA was observed in the Native PAGE analysis (Figure S1b). Collectively, these results suggest that Aβ amyloid formation was not inhibited by PAA. As discussed above, the ThT intensity of Aβ amyloids was slightly decreased by the incubation with PAA (Figure 1a), possibly due to the binding of PAA to the amyloids. Thus, it is plausible that PAA also binds to Aβ amyloids more efficiently when it exists before the incubation, and that it hinders binding of ThT to Aβ amyloids. Interestingly, no change in the electrophoretic mobility was observed for Aβ monomers incubated with PAA in the Native PAGE analysis (Figure S1b), supporting that PAA does not bind to Aβ monomers. In the SEM observation, morphologically similar amyloids were observed (Figure 4a and Figure 8c), indicating that PAA itself did not affect the structure of Aβ amyloids as in the case of incubation with PAA after amyloid formation (Figure 4b). Importantly, inhibition in the binding of antibodies (6E10 and 4G8) to Aβ amyloids was also observed for the Aβ samples incubated with PAA (Figure 8d). These results correspond with those using the Aβ samples incubated with PAA after the formation of amyloids, as described above.

## 3. Materials and Methods

### 3.1. Materials

Lyophilized, synthetic Aβ42 was obtained from Peptide Institute Inc. (Osaka, Japan). ThT was obtained from Sigma Chemical Co. (St. Louis, MO, USA). The PAA (PAA01, mean molecular weight, 1600) was obtained from Nittobo Medical (Tokyo, Japan). Anti-Aβ antibodies (6E10 and 4G8) were purchased from BioLegend (San Diego, CA, USA). The Cy3 monofunctional NHS ester fluorescent dye was obtained from GE Healthcare (Chicago, IL, USA).

### 3.2. Formation of Aβ42 Amyloid and Effect of PAA

The seed-free Aβ42 stock solution was prepared as previously described [34]. For the formation of Aβ amyloids, 50 µM Aβ42 was incubated in a phosphate buffer (50 mM phosphate buffer (pH 7.0) and 200 mM NaCl) at 37 °C for 24 h. To examine the effect of PAA on Aβ amyloids, 25 µM Aβ amyloid samples were incubated with PAA at various concentrations (0–50 µM) in the phosphate buffer at room temperature for 5 min for the following analyses. We confirmed that incubation for 5 min is sufficient since the ThT intensity of the samples incubated with PAA for 24 h was similar (Figure S3). To examine the effect of PAA on amyloid formation, 25 µM Aβ monomer samples were incubated with PAA at various concentrations (0–50 µM) in 50 mM phosphate buffer (pH 7.0) at 37 °C for 24 h.

### 3.3. ThT Assay

Aβ amyloids were estimated using a ThT fluorescence assay as described previously [35]. The fluorescent intensity of 2.5 μM Aβ42 samples including 20 μM ThT in the phosphate buffer was measured (excitation, 420 nm; emission, 490 nm) using a microplate reader (BioTek, Winooski, VT, USA). The fluorescence of the 20 μM ThT solution was used for background subtraction. The average values of three wells were calculated. Statistical analysis was performed using Student’s t-test.

### 3.4. Fluorescence Microscopy

Cy3-modified PAA (Cy3-PAA) was prepared as follows. Cy3 dye (2 µM) was incubated with 200 µM PAA in a 100 mM phosphate buffer, pH 8.0, at room temperature for 2 h. Then, 100 µM Cy3-PAA was incubated with 25µM Aβ amyloid at room temperature for 2 h. A higher concentration of PAA was used here to ensure the binding of Cy3-PAA to Aβ amyloids. To remove the unbound Cy3-PAA, the sample was centrifuged at 21,600× *g* rpm for 1 h, by which time approximately 90% of proteins were collected. The precipitated sample was redispersed in water to obtain the 25 µM Aβ samples. The Aβ42 amyloid incubated with 1 µM Cy3 dye that was pre-incubated in a 100 mM phosphate buffer, pH 8.0, at room temperature for 2 h was used as a negative control. Fluorescence images were captured with a fluorescence microscope (Axioplan2, Zeiss, Oberkochen, Germany) equipped with a DP74 CCD camera (Olympus, Tokyo, Japan) and a UPlanApo 20× objective lens (Olympus) using CellSens software (Version 3. 1. 1) (Olympus). Fluorescent intensities of the fluorescent spots were analyzed using ImageJ software (ver. 1.54) [36]. The average values of the spots from three individual images for each sample were used. Statistical analysis was performed using Student’s t-test.

### 3.5. Tyrosine Fluorescence

Aβ amyloids (8.3 µM) were incubated with PAA at various concentrations (0–16.7 µM). Fluorescence spectra were obtained using a spectrofluorometer (FP-8600, Jasco, Tokyo, Japan) with excitation and emission wavelengths as discussed in the text. For the Stern–Volmer plot, the integrated fluorescence signal (285–360 nm) derived from tyrosine excited in the 270–280 nm range was used. The average values of three independent measurements were used.

### 3.6. Scanning Electron Microscope (SEM)

SEM analysis was performed as described previously [35]. The 5 μM Aβ samples diluted in water were placed on a silicon wafer and allowed to air-dry. The samples were observed at 5000 magnifications at a working distance of 10.0 mm using a field-emission scanning electron microscope (FE-SEM, JSM7001FA, JEOL, Tokyo, Japan) operated at an acceleration voltage of 15 kV.

### 3.7. Dot Blot Assay

Dot blot assays were performed as previously described [37]. The 25 μM Aβ samples (2 μL) were spotted onto a nitrocellulose membrane (0.22 μm, GE Healthcare Life Sciences, Buckinghamshire, UK). After blocking with 5% skim milk in Tris-buffered saline containing 0.01% Tween20 (0.01% TBST) for 1 h at room temperature, the membrane was incubated with anti-Aβ (6E10, 1:5000) or (4G8, 1:4000) at 4 °C overnight, followed by a secondary anti-mouse IgG antibody (1:5000 in TBST) for 1 h at room temperature. Proteins were visualized using Clarity Western ECL substrate (Bio-Rad, Hercules, CA, USA) on an LAS4000 mini luminescent image analyzer (Fujifilm, Tokyo, Japan). The average intensity of three dots was used.

### 3.8. Cytotoxicity Assay

Cell viability was determined using a 3-(4,5-dimethylthiazol- 2-yl)-2,5-diphenyltetrazolium bromide (MTT) assay (Cell Proliferation Kit. Roche, Basel, Switzerland) as previously described [7,16]. PC12 cells (a clonal line of rat pheochromocytoma) were cultured in RPMI 1640 medium (containing 5% FBS, 10% horse serum, 1% penicillin–streptomycin) in poly-D-Lysine (PDL)-coated dishes at 37 °C, 5% CO_2_. Cells (80 μL) were plated in PDL-coated 96-well plates at a density of 20,000 cells/well and incubated overnight. Then, 20 μL Aβ samples diluted with PBS to the desired concentrations were added to the corresponding wells, including 80 μL of the PC12 cell suspension, and incubated for 24 h at 37 °C. The final Aβ42 concentrations were 0.05 μM (with/without 0.01 μM PAA) and 0.1 μM (with/without 0.02 μM PAA), at which concentrations the fibrils were toxic while the monomers were nontoxic to the cells. Then, 10 μL of the MTT reagent was added to each well, and the absorbance at 562 nm was measured using the plate reader (BioTek). A monomer sample was used as a negative control immediately after being dissolved in PBS. The viability of cells exposed to PBS was used as the 100% viability control. Each data point represented the average of three wells.

### 3.9. Measurement of the Protein Concentration in the Supernatant

To evaluate the amount of Aβ amyloid, the Aβ42 samples incubated with PAA were centrifuged at 15,000 rpm at 4 °C for 60 min. Protein concentrations in the supernatant were analyzed using a bicinchoninic acid (BCA) assay (Pierce™ BCA Protein Assay Kit). Bovine serum albumin (BSA) samples containing the corresponding concentrations of PAA were used as standards.

## 4. Conclusions

In this study, we investigated the effect of PAA on Aβ amyloids and demonstrated that PAA could bind to Aβ amyloids using a variety of techniques: ThT and tyrosine-quenching experiments along with microscopic techniques. Our results suggest that PAA can be used as a coating reagent for amyloids. Polymorphism in the amyloid structure has been reported. For example, a different twist structure was observed between Aβ amyloids formed in an AD patient’s brain and those formed in vitro [38]. It has been shown that Aβ oligomers can bind to NH_2_-modified surfaces [33], suggesting that PAA could be used for the coating of Aβ oligomers. It is also plausible that other polymers with amino groups can interact with Aβ amyloids. Further studies are necessary to confirm these hypotheses. In this study, it was shown that the binding of antibodies to Aβ amyloids was inhibited by PAA. The binding of ThT dye was also slightly inhibited by PAA. These probes have been used for amyloid detection; therefore, our results also serve as a possible indication for the analysis of functional amyloids coated with various polymers.

## Data Availability

Data are contained within the article and Supplementary Materials.

## Supplementary Materials

The following supporting information can be downloaded at https://www.mdpi.com/article/10.3390/ijms25063112/s1.
