# Supplementary material for: Polyallylamine Binds to Aβ Amyloid and Inhibits Antibody Recognition
Source: Int J Mol Sci. 2024 Mar 7;25(6):3112. doi: 10.3390/ijms25063112 (PMC10970444; doi:10.3390/ijms25063112)
Supplement: Supplementary file 1 [file ijms-25-03112-s001.zip › ijms-2822594-supplementary.pdf]

Supplementary Information

Polyallylamine binds to Aβ amyloid and inhibits antibody recognition

Yusuke Tsuchie [1,†], Soichiro Kusuda [2,†], Haruka Kawabe [1], Wakako Mori [1], Mikael Lindgren [2,3], Yutaka Watanabe [2] and Tamotsu Zako [1,2*]

[1]Department of Chemistry and Biology, Graduate School of Science and Engineering, Ehime University, 2-5 Bunkyo, Matsuyama, Ehime 790-8577, Japan zako.tamotsu.us@ehime-u.ac.jp (T.Z.)
[2]Department of Chemistry, Faculty of Science, Ehime University, 2-5 Bunkyo, Matsuyama, Ehime 790-8577, Japan
[3]Department of Physics, Faculty of Natural Sciences, Norwegian University of Science and Technology (NTNU), Gløshaugen, Realfagsbygget, Trondheim 7490, Norway
**Correspondence: zako.tamotsu.us@ehime-u.ac.jp*
[†]These authors contributed equally to this work.

Table of Contents

Figure S1 Native PAGE analysis of the Aβ samples incubated with PAA.

Figure S2 Dot blot assay of Aβ amyloid with or without PAA.

Figure S3 ThT assay of Aβ amyloid incubated with PAA for 5 min or 24 h.

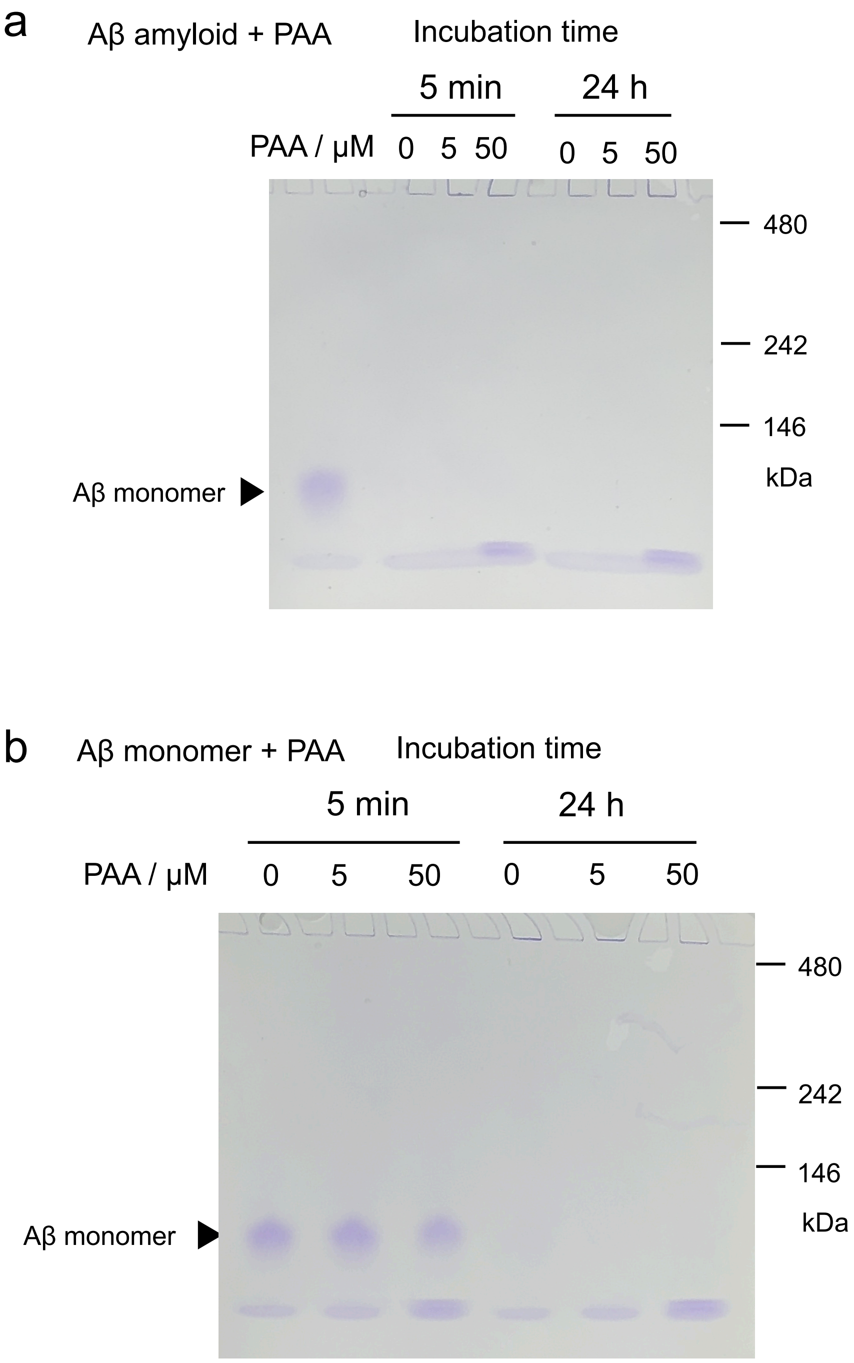

Figure S1. Native PAGE analysis of the Aβ samples incubated with PAA. **a)** Native PAGE analysis of Aβ amyloids incubated with PAA. The Aβ amyloids samples (25 μM) were incubated in the absence or presence of 5 and 50 μM PAA for 5 min, and the samples (13.5 μL) mixed with a sample buffer were applied to an 8% native gel and run at a current of 20 mA. **b)** Native PAGE analysis of the Aβ monomer samples incubated with PAA. Aβ monomer samples (25 μM) were incubated in the absence or presence of 5 and 50 μM PAA for 24 h, and the samples (13.5 μL) mixed with a sample buffer were applied to an 8% native gel. The Aβ monomer samples incubated with PAA for 5 min were shown as control samples. The proteins were stained by 0.1% (w/v) Coomassie Brilliant Blue (CBB) solution. The marker proteins are shown as molecular weight markers in kDa.

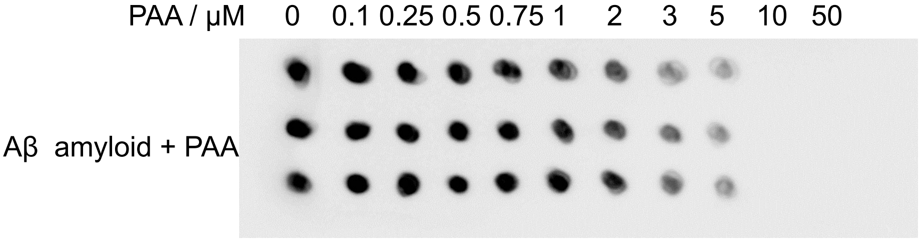

Figure S2. Dot blot assay of Aβ amyloid with or without PAA. Dot blot images of Aβ amyloid incubated with 0–50 µM PAA for 5 min using 6E10 antibody.

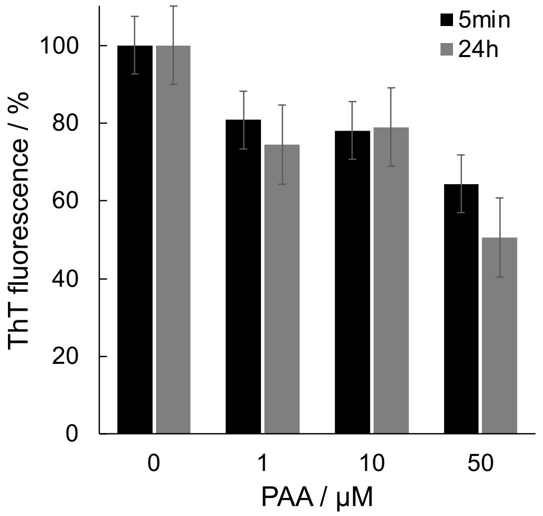

Figure S3. ThT assay of Aβ amyloid incubated with PAA for 5 min or 24 h. The Aβ amyloid samples (25 µM) were incubated with 0-50 µM PAA for 5 min or 24 h at room temperature before ThT assay. The fluorescent intensity of 2.5 µM Aβ amyloid samples including 20 µM ThT in the phosphate buffer was measured (excitation, 420 nm; emission, 490 nm) using a microplate reader. The average values of three wells were calculated. The intensity of the samples without PAA was normalized as 100%.
